# Pediatric Hospitalizations at Rural and Urban Teaching and Nonteaching Hospitals in the US, 2009-2019

**DOI:** 10.1001/jamanetworkopen.2023.31807

**Published:** 2023-09-01

**Authors:** JoAnna K. Leyenaar, Seneca D. Freyleue, Mary Arakelyan, David C. Goodman, A. James O’Malley

**Affiliations:** 1 Department of Pediatrics, Dartmouth Health Children’s, Lebanon, New Hampshire; 2 The Dartmouth Institute for Health Policy & Clinical Practice, Geisel School of Medicine at Dartmouth, Lebanon, New Hampshire; 3 Department of Biomedical Data Science, Geisel School of Medicine at Dartmouth, Lebanon, New Hampshire

## Abstract

**Importance:**

National analyses suggest that approximately 1 in 5 US hospitals closed their pediatric units between 2008 and 2018. The extent to which pediatric hospitalizations at general hospitals in rural and urban communities decreased during this period is not well understood.

**Objective:**

To describe changes in the number and proportion of pediatric hospitalizations and costs at urban teaching, urban nonteaching, and rural hospitals vs freestanding children’s hospitals from 2009 to 2019; to estimate the number and proportion of hospitals providing inpatient pediatric care; and to characterize changes in clinical complexity.

**Design, Setting, and Participants:**

This study is a retrospective cross-sectional analysis of the 2009, 2012, 2016, and 2019 Kids’ Inpatient Database, a nationally representative data set of US pediatric hospitalizations among children younger than 18 years. Data were analyzed from February to June 2023.

**Exposures:**

Pediatric hospitalizations were grouped as birth or nonbirth hospitalizations. Hospitals were categorized as freestanding children’s hospitals or as rural, urban nonteaching, or urban teaching general hospitals.

**Main Outcomes and Measures:**

The primary outcomes were annual number and proportion of birth and nonbirth hospitalizations and health care costs, changes in the proportion of hospitalizations with complex diagnoses, and estimated number and proportion of hospitals providing pediatric care and associated hospital volumes. Regression analyses were used to compare health care utilization in 2019 vs that in 2009.

**Results:**

The data included 23.2 million (95% CI, 22.7-23.6 million) weighted hospitalizations. From 2009 to 2019, estimated national annual pediatric hospitalizations decreased from 6 425 858 to 5 297 882, as birth hospitalizations decreased by 10.6% (95% CI, 6.1%-15.1%) and nonbirth hospitalizations decreased by 28.9% (95% CI, 21.3%-36.5%). Concurrently, hospitalizations with complex chronic disease diagnoses increased by 45.5% (95% CI, 34.6%-56.4%), and hospitalizations with mental health diagnoses increased by 78.0% (95% CI, 61.6%-94.4%). During this period, the most substantial decreases were in nonbirth hospitalizations at rural hospitals (4-fold decrease from 229 263 to 62 729) and urban nonteaching hospitals (6-fold decrease from 581 320 to 92 118). In 2019, birth hospitalizations occurred at 2666 hospitals. Nonbirth pediatric hospitalizations occurred at 3507 hospitals, including 1256 rural hospitals and 843 urban nonteaching hospitals where the median nonbirth hospitalization volumes were fewer than 25 per year.

**Conclusions and Relevance:**

Between 2009 and 2019, the largest decreases in pediatric hospitalizations occurred at rural and urban nonteaching hospitals. Clinical and policy initiatives to support hospitals with low pediatric volumes may be needed to maintain hospital access and pediatric readiness, particularly in rural communities.

## Key Points

**Question:**

How have pediatric inpatient hospitalizations at US urban and rural general hospitals and freestanding children’s hospitals changed from 2009 to 2019?

**Findings:**

In this cross-sectional analysis of a national data set representing an estimated 23.2 million inpatient pediatric hospitalizations from 2009 to 2019, birth hospitalizations decreased by 10.6%, whereas nonbirth hospitalizations decreased by 28.9%. The largest decreases were at urban nonteaching and rural hospitals, most of which had 25 or fewer nonbirth pediatric hospitalizations in 2019.

**Meaning:**

These findings suggest that clinical and policy initiatives to support hospitals with low pediatric volumes may be needed to maintain pediatric hospital access and capabilities, particularly in rural communities.

## Teaser

This cross-sectional study examines changes in pediatric hospitalizations and related costs at urban teaching, urban nonteaching, and rural hospitals vs freestanding children’s hospitals in the US from 2009 to 2019.

## Introduction

In 2019, 5.2 million pediatric hospitalizations in the US incurred costs in excess of $46 billion, making inpatient care among the most costly components of pediatric health care.^1,2^ Although pediatric hospitalization costs have increased substantially over the last decade, reimbursement for these stays represents a small fraction of most hospitals’ total revenue.^3,4,5^ An analysis of approximately 4400 hospitals surveyed annually by the American Hospital Association (AHA) found that less than half had pediatric units, with an almost 20% decrease from 2008 to 2018.^6^ These pediatric unit closures parallel trends in increasing regionalization of care, with a growing proportion of children who seek care at general hospital emergency departments being transferred to other hospitals for admission.^7,8,9,10^

Although pediatric unit closures have received substantial media attention, the extent to which children continue to be hospitalized in general hospitals, including rural hospitals and urban teaching and nonteaching centers, is not well understood.^11,12^ Studies conducted at freestanding children’s hospitals (FCHs), all of which are located in urban communities, have demonstrated increasing medical complexity and intensive care unit admissions over time.^13,14,15,16,17^ Patterns of care at general hospitals are less well characterized, although several national studies have documented trends in closure of hospitals’ obstetric services.^18,19,20^ To address this knowledge gap, this study describes changes in the number and proportion of birth and nonbirth pediatric hospitalizations and health care costs at urban teaching, urban nonteaching, and rural hospitals compared with FCHs from 2009 to 2019; estimates the number and proportion of hospitals providing inpatient pediatric care; and characterizes changes in resource utilization, payer mix, and clinical complexity during the period.

## Methods

### Study Design and Data Sources

We conducted a retrospective cross-sectional analysis of the 2009, 2012, 2016, and 2019 Kids’ Inpatient Database (KID), a nationally representative data set typically published every 3 years by the Healthcare Cost and Utilization Project (HCUP), Agency for Healthcare Research and Quality.^21^ The KID includes short-term, nonfederal, general, and specialty hospitals and excludes rehabilitation, long-term, and psychiatric hospitals.^21^ In 2009 and 2012, 44 states contributed to the KID; in 2016 and 2019, the KID included data from 47 and 49 states, respectively. Each data set represents a calendar year of inpatient hospitalization data for patients aged 20 years and younger, including a 10% systematic random sample of uncomplicated birth hospitalizations and an 80% sample of other birth and pediatric discharges. Data sets also include discharge weights for each hospitalization based on the universe of community nonrehabilitation hospitals surveyed annually by the AHA.

Birth and nonbirth hospitalizations among children and adolescents younger than 18 years were included in this analysis, because those aged 18 years or older are recognized as adults in most states and are typically admitted to adult beds at general hospitals.^22^ We excluded 4766 hospitalizations (<0.1%) that were missing a principal *International Classification of Disease, Ninth Revision* (*ICD-9*) or *International Statistical Classification of Diseases and Related Health Problems, Tenth Revision* (*ICD-10*) discharge diagnosis.

HCUP databases are limited data sets, and the Dartmouth-Health institutional review board determined that this study did not constitute human participants research; therefore, informed consent was not needed, in accordance with 45 CFR §46.^23^ The methods and results reported here adhere with the Strengthening the Reporting of Observational Studies in Epidemiology (STROBE) reporting guideline for cross-sectional studies and HCUP data use agreement requirements.^24^

### Hospital Characteristics

We categorized hospitals into 4 mutually exclusive types: rural, urban nonteaching, urban teaching (together comprising general hospitals), and FCHs. Given the rarity of rural teaching hospitals, rural hospitals are not stratified according to teaching status in the KID.^21^ Teaching hospitals are defined as having Accreditation Council for Graduate Medical Education–approved residency programs, membership in the Council of Teaching Hospitals, or a ratio of full-time interns and residents to beds of greater than or equal to 0.25.^21^ FCHs are assigned to a separate stratum in the KID. We estimated the annual number of birth and nonbirth hospitalizations at each hospital, applying weights to generate hospital-level estimates. For example, for nonbirth hospitalizations, the number of hospitalizations per hospital was determined by multiplying the number of encounters by 1.25, because the KID includes an 80% sample of these discharges.^21^ Additional hospital characteristics included geographic region based on US Census Regions and hospital ownership.

### Hospitalization Characteristics

For each hospitalization, we examined patient age at admission in years, binary sex (male or female as reported in the KID), race and ethnicity reported to HCUP by partner organizations and collected according to each hospital’s standard operating procedures,^25^ expected primary payer, and median household income of each patient’s zip code (in quartiles). Race and ethnicity were reported using the variable labels provided by HCUP, because of the disparities in hospitalization rates associated with race and ethnicity.^26,27,28^

Birth hospitalizations were identified using the KID in-hospital birth indicator variable and were categorized as uncomplicated or complicated according to Diagnosis Related Groups.^21^ The Pediatric Medical Complexity Algorithm was applied to identify nonbirth hospitalizations with complex chronic disease diagnoses, defined as those with malignant neoplasms, progressive conditions, or chronic diseases in 2 or more body systems.^29,30,31^ Similarly, the Children with Disabilities Algorithm was applied to identify those with disabilities, and the Child and Adolescent Mental Health Disorders Classification System was used to identify those with 1 or more mental health diagnoses in any *ICD-9* or *ICD-10* position.^32,33^

For each hospitalization, we determined length of stay (LOS) in days, total costs adjusted to 2019 dollars, whether the hospitalization followed a transfer from another acute care hospital (which includes transfers from their emergency departments), and whether the hospitalization resulted in a transfer to another short-term hospital.^21^ Transfers from other hospitals were limited to nonbirth hospitalizations. Costs were examined using data on total charges (total amount billed), which were converted to costs (expenses incurred in the provision of care) using HCUP cost-to-charge ratios.^34^ We adjusted costs from 2009 to 2016 to represent 2019 dollars using the Personal Consumption Expenditures index using a guide published by the Agency for Healthcare Research and Quality.^35^

### Statistical Analysis

After applying the KID survey weights to generate national estimates, we summarized sociodemographic and hospital characteristics using counts and percentages with associated 95% CIs. Using point and interval estimators for survey data, we determined the sampling probability-weighted number and proportion of birth and nonbirth hospitalizations and inpatient health care costs at each hospital type each year and plotted these against year with associated 95% CIs. To characterize changes in clinical complexity over time, we determined the proportion of complicated birth hospitalizations at each hospital type, as well as the proportion of nonbirth hospitalizations with 1 or more complex chronic disease diagnosis, disability diagnosis, and mental health diagnosis. Taylor series methods were used to approximate SEs and construct 95% CIs,^36^ accounting for clustering at the level of the hospital with the hospitalization treated as the primary sampling unit. Regression analyses were used to compare health care utilization in 2019 compared with 2009, using Poisson regression for LOS, linear regression for costs, and logistic regression for interfacility transfers, clustering hospitalizations within hospitals and computing SEs that were robust to misspecification of the within-hospital covariance matrix by using survey design estimators.

Analyses were conducted using R statistical software version 4.1.3 (R Project for Statistical Computing), SAS statistical software version 9.4 (SAS Institute), and Python programming language version 3.8.10 (Python Software Foundation). The threshold for statistical significance was 2-sided *P* < .05. We reported the amount of missing data in the tables and figures and used pairwise deletion as appropriate. Data were analyzed from February to June 2023.

## Results

### Hospitalization Characteristics

Across the 4 years of data, the KID included 12 809 624 million unweighted hospitalizations (3 407 146 in 2009, 3 195 782 in 2012, 3 117 413 in 2016, and 3 089 283 in 2019) and 23.2 million (95% CI, 22.7-23.6 million) weighted hospitalizations. From 2009 to 2019, the estimated total annual pediatric hospitalizations decreased from 6 425 858 to 5 297 882, with birth hospitalizations decreasing by 10.6% (95% CI, 6.1%-15.1%) and nonbirth hospitalizations decreasing by 28.9% (95% CI, 21.3%-36.5%). Birth hospitalizations accounted for a larger proportion of pediatric hospitalizations over time, comprising 62.1% (95% CI, 60.6%-63.6%) of hospitalizations in 2009 and 67.3% (95% CI, 65.5%-69.2%) in 2019.

The distribution of sociodemographic characteristics was similar across years (Table 1). Most hospitalizations occurred among urban-residing children (2019, 4 560 631 children; 91.3%, 95% CI, 90.8%-91.9%), among boys (2019, 2 715 748 boys; 51.3%, 95% CI, 51.2%-51.4%), and among children covered by Medicaid (2019, 2 531 983 children; 47.9%, 95% CI, 46.8%-48.9%). Hospital ownership and the geographic distribution of hospitalizations were similar across years.

**Table 1.** Sociodemographic, Clinical, and Hospital Characteristics of Pediatric Hospitalizations, Weighted National Estimates, 2009-2019

Characteristic	Hospitalizations, No. (%) [95% CI]			
	2009	2012	2016	2019
Sociodemographic and clinical characteristics				
Reason for hospitalization^a^	6 425 858	5 857 430	5 602 612	5 297 882
Birth hospitalization	3 992 661 (62.1) [60.6-63.6]	3 770 047 (63.8) [62.0-65.5]	3 770 047 (67.3) [65.4-69.1]	3 567 900 (67.3) [65.5-69.2]
Nonbirth hospitalization	2 433 197 (37.9) [36.3-39.4]	2 121 092 (36.2) [34.5-38.0]	1 832 565 (32.7) [30.9-34.6]	1 729 982 (32.7) [30.8-34.5]
Nonbirth hospitalization age^a^	2 412 032	2 112 107	1 832 565	1 729 982
Infant, ≤1 y	619 317 (25.7) [24.9-26.4]	532 596 (25.2) [24.7-25.8]	480 987 (26.2) [25.5-27.0]	456 697 (26.4) [25.6-27.1]
Early childhood, 1-4 y	564 712 (23.4) [23.0-23.8]	481 646 (22.8) [22.4-23.2]	386 949 (21.1) [20.6-21.6]	372 013 (21.5) [21.0-22.0]
Middle childhood, 5-10 y	412 906 (17.1) [16.8-17.4]	374 184 (17.7) [17.4-18.0]	324 628 (17.7) [17.3-18.1]	289 119 (16.7) [16.3-17.1]
Early adolescence, 11-14 y	317 565 (13.2) [12.9-13.5]	306 054 (14.5) [14.2-14.8]	277 864 (15.2) [14.8-15.6]	279 954 (16.2) [15.8-16.6]
Later adolescence, 15-17 y	497 531 (20.6) [20.0-21.3]	417 627 (19.8) [19.1-20.4]	362 137 (19.8) [19.1-20.4]	332 199 (19.2) [18.6-19.8]
Sex^a^	6 371 933	5 855 167	5 599 895	5 295 596
Female	3 109 948 (48.8) [48.7-49.0]	2 857 198 (48.8) [48.7-48.9]	2 733 657 (48.8) [48.7-49.0]	2 579 848 (48.7) [48.6-48.8]
Male	3 261 985 (51.2) [51.0-51.3]	2 997 969 (51.2) [51.1-51.3]	2 866 238 (51.2) [51.0-51.3]	2 715 748 (51.3) [51.2-51.4]
Race and ethnicity^a^^,^^b^	5 378 812	5 355 333	5 050 292	4 862 344
Hispanic	1 242 253 (23.1) [21.6-24.6]	1 127 385 (21.1) [19.7-22.4]	1 063 687 (21.1) [19.7-22.4]	1 017 154 (20.9) [19.6-22.2]
Non-Hispanic Asian or Pacific Islander	206 950 (3.8) [3.5-4.2]	244 904 (4.6) [4.1-5.1]	265 395 (5.3) [4.7-5.8]	249 679 (5.1) [4.7-5.6]
Non-Hispanic Black	817 489 (15.2) [14.3-16.1]	834 403 (15.6) [14.7-16.4]	790 402 (15.7) [14.9-16.4]	774 584 (15.9) [15.1-16.7]
Non-Hispanic Native American	51 659 (1.0) [0.8-1.2]	49 927 (0.9) [0.8-1.1]	41 935 (0.8) [0.7-1.0]	40 505 (0.8) [0.7-1.0]
Non-Hispanic White	2 756 068 (51.2) [49.8-52.7]	2 754 950 (51.4) [50.0-52.9]	2 569 723 (50.9) [49.5-52.3]	2 438 223 (50.1) [48.8-51.5]
Other^c^	304 393 (5.7) [5.1-6.2]	343 764 (6.4) [5.7-7.1]	319 150 (6.3) [5.7-6.9]	342 199 (7.0) [6.4-7.6]
Rurality of residence^a^^,^^d^	5 892 035	5 495 004	5 274 741	4 993 692
Urban residing	5 240 714 (88.9) [88.3-89.6]	4 920 353 (89.5) [89.0-90.1]	4 814 531 (91.3) [90.8-91.8]	4 560 631 (91.3) [90.8-91.9]
Rural residing	651 321 (11.1) [10.4-11.7]	574 651 (10.5) [9.9-11.0]	460 210 (8.7) [8.2-9.2]	433 061 (8.7) [8.1-9.2]
Primary payer^a^	6 415 844	5 842 474	5 595 232	5 290 584
Medicaid	3 037 431 (47.3) [46.3-48.4]	2 847 808 (48.7) [47.7-49.8]	2 716 107 (48.5) [47.5-49.6]	2 531 983 (47.9) [46.8-48.9]
Medicare	11 643 (0.2) [0.1-0.2]	20 690 (0.4) [0.3-0.5]	20 103 (0.4) [0.3-0.5]	14 093 (0.3) [0.2-0.3]
Private insurance	2 909 890 (45.4) [44.2-46.5]	2 553 164 (43.7) [42.6-44.8]	2 458 897 (43.9) [42.9-45.0]	2 339 106 (44.2) [43.1-45.3]
Other	204 109 (3.2) [2.9-3.5]	217 058 (3.7) [3.3-4.1]	181 294 (3.2) [2.9-3.6]	161 400 (3.1) [2.7-3.4]
Self-pay	252 771 (3.9) [3.6-4.3]	203 754 (3.5) [3.2-3.8]	218 830 (3.9) [3.7-4.2]	244 002 (4.6) [4.3-4.9]
Median income at zip code^a^^,^^e^	6 290 954	5 745 305	5 537 021	5 245 645
Quartile 1	1 885 135 (30.0) [28.8-31.2]	1 727 376 (30.1) [28.9-31.3]	1 645 728 (29.7) [28.6-30.9]	1 527 091 (29.1) [27.9-30.3]
Quartile 2	1 639 050 (26.1) [25.3-26.8]	1 415 755 (24.6) [23.9-25.4]	1 359 819 (24.6) [23.8-25.3]	1 284 575 (24.5) [23.7-25.3]
Quartile 3	1 500 658 (23.9) [23.1-24.6]	1 375 434 (23.9) [23.2-24.6]	1 348 986 (24.4) [23.7-25.0]	1 307 311 (24.9) [24.3-25.6]
Quartile 4	1 266 111 (20.1) [18.9-21.3]	1 226 740 (21.4) [20.1-22.6]	1 182 488 (21.4) [20.1-22.6]	1 126 668 (21.5) [20.2-22.7]
Hospital characteristics				
Hospital region^a^	6 425 858	5 857 430	5 602 612	5 297 882
Midwest	1 374 224 (21.4) [19.8-23.0]	1 265 681 (21.6) [19.9-23.3]	1 205 089 (21.5) [19.7-23.3]	1 137 302 (21.5) [19.6-23.4]
Northeast	1 069 211 (16.6) [15.1-18.2]	995 169 (17.0) [15.3-18.7]	928 142 (16.6) [14.8-18.3]	867 253 (16.4) [14.5-18.2]
South	2 481 168 (38.6) [36.6-40.7]	2 236 154 (38.2) [36.1-40.3]	2 170 813 (38.7) [36.5-41.0]	2 076 360 (39.2) [36.9-41.5]
West	1 501 255 (23.4) [21.6-25.1]	1 360 426 (23.2) [21.4-25.1]	1 298 568 (23.2) [21.2-25.1]	1 216 967 (23.0) [21.0-25.0]
Hospital ownership^a^^,^^f^	6 071 362	5 857 430	5 602 612	5 297 882
Private, nonprofit	4 479 846 (73.8) [71.6-76.0]	4 319 268 (73.7) [72.2-75.3]	4 173 932 (74.5) [72.9-76.1]	4 026 302 (76.0) [74.5-77.5]
Government, nonfederal	839 928 (13.8) [12.0-15.6]	765 084 (13.1) [12.1-14.1]	691 735 (12.3) [11.4-13.3]	659 520 (12.4) [11.4-13.5]
Private, investor owned	751 588 (12.4) [10.9-13.8]	773 078 (13.2) [11.9-14.5]	736 946 (13.2) [11.9-14.4]	612 060 (11.6) [10.4-12.7]
Hospital location and teaching status^a^^,^^g^^,^^h^	6 187 039	5 857 430	5 602 612	5 297 882
General hospitals	5 625 000 (90.9) [89.3-92.5]	5 279 244 (90.1) [88.3-92.0]	5 009 817 (89.4) [87.4-91.4]	4 695 094 (88.6) [86.6-90.6]
Rural hospitals	703 301 (11.4) [10.7-12.0]	604 416 (10.3) [9.7-10.9]	453 319 (8.1) [7.6-8.6]	395 564 (7.5) [7.0-7.9]
Urban nonteaching hospitals	2 304 866 (37.3) [35.6-38.9]	1 811 307 (30.9) [29.5-32.4]	1 107 002 (19.8) [18.6-20.9]	710 075 (13.4) [12.4-14.4]
Urban teaching hospitals	2 616 833 (42.3) [40.5-44.1]	2 863 521 (48.9) [47.1-50.7]	3 449 496 (61.6) [59.7-63.5]	3 589 455 (67.8) [65.8-69.7]
Freestanding children’s hospitals	562 039 (9.1) [7.5-10.7]	578 186 (9.9) [8.0-11.7]	592 794 (10.6) [8.6-12.6]	602 788 (11.4) [9.4-13.4]

^a^ Data in this row are weighted national estimates of nonmissing data; variables with 3.0% or more missing data are also indicated with footnotes.

^b^ Hospitalizations with missing values are as follows: 2009, 1 047 046 (16.3%); 2012, 502 097 (8.6%); 2016, 552 320 (9.9%); and 2019, 435 538 (8.2%).

^c^ Other race is a category used by the Kids’ Inpatient database; additional description about this variable at the national level is not provided.

^d^ Hospitalizations with missing values are as follows: 2009, 533 823 (8.3%); 2012, 362 426 (6.2%); 2016, 327 871 (5.9%); and 2019, 304 190 (5.7%).

^e^ Quartile ranges for median household income at home zip code vary annually (eg, quartile 1 encompasses median household incomes of less than $40 000 in 2009 and less than $48 000 in 2019). Details are available in the Healthcare Cost and Utilization Project data dictionaries.

^f^ In 2009, there were 354 496 hospitalizations (5.5%) with missing values.

^g^ Starting in 2014, more hospitals were categorized as urban teaching hospitals because there was an increase in facilities with approved residency programs in the American Hospital Association Annual Survey, from which hospital characteristics are derived. The Accreditation Council for Graduate Medical Education became the primary body for residency approval around this time.

^h^ In 2009, there were 238 819 hospitalizations (3.7%) with missing data.

### Changes in Hospitalization Settings and Costs Over Time

**Figure 1.**
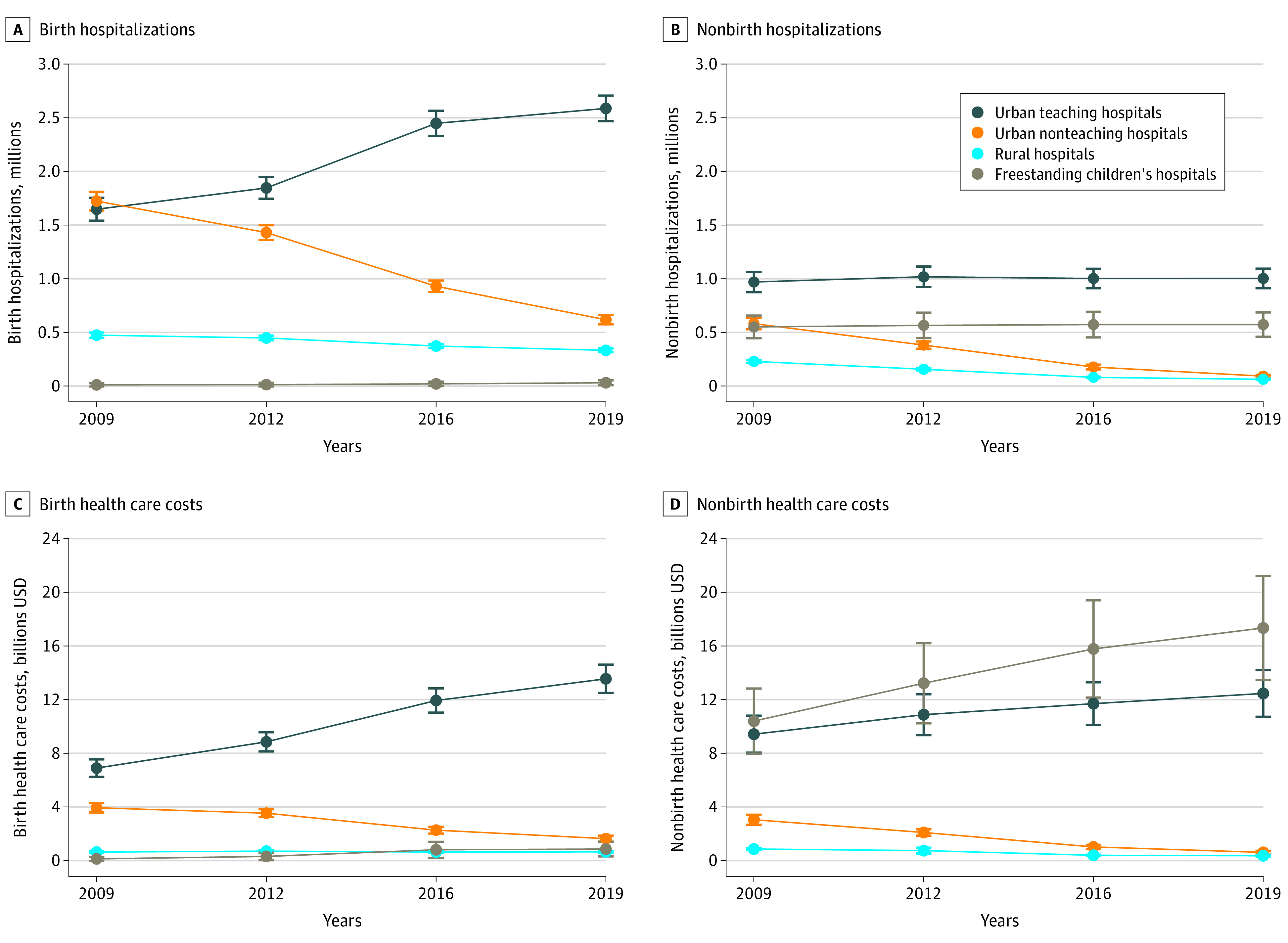
Birth and Nonbirth Pediatric Hospitalizations and Total Hospitalization Costs at Rural Hospitals, Urban Nonteaching and Teaching Hospitals, and Freestanding Children’s Hospitals, 2009-2019 Data are weighted national estimates. Error bars denote 95% CIs. The hospital type was missing for 238 818 hospitalizations (3.7%) in 2009.

Birth hospitalizations at rural hospitals and urban nonteaching hospitals decreased significantly from 2009 to 2019, whereas hospitalizations at urban teaching hospitals increased; all changes were monotonic over time (Figure 1; eTable 1 in Supplement 1). During this period, the proportion of birth hospitalizations at rural hospitals decreased by approximately 25%, from 12.3% (95% CI, 11.6%-13.0%) to 9.3% (95% CI, 8.8%-9.9%). At rural hospitals, there was an almost 4-fold decrease in nonbirth pediatric hospitalizations, from 229 263 (9.8% of nonbirth hospitalizations; 95% CI, 9.0% to 10.7% of nonbirth hospitalizations) in 2009 to 62 729 (3.6% of nonbirth hospitalizations; 95% CI, 3.2% to 4.1% of nonbirth hospitalizations) in 2019. At urban nonteaching hospitals, nonbirth hospitalizations decreased by a factor of 6, from 581 320 (24.9% of hospitalizations; 95% CI, 22.7% to 27.2% of hospitalizations) in 2009 to 92 118 (5.3% of hospitalizations; 95% CI, 4.5% to 6.1% of hospitalizations) in 2019. There was a concurrent increase of 33 021 nonbirth hospitalizations (95% CI, −98 395 to 164 437 hospitalizations) at urban teaching hospitals, reflecting a relative increase of 3.4% (95% CI, −10.4% to 17.2%) and of 21 717 hospitalizations (95% CI, −133 283 to 176 717 hospitalizations) at FCHs, reflecting a relative increase of 3.9% (95% CI, −24.8% to 32.7%).

From 2009 to 2019, birth hospitalization costs increased from an estimated $11.6 billion to $16.7 billion, and the probability distribution of these costs changed significantly, with the largest decrease (−$2.3 billion; 95% CI, −$2.7 billion to −$1.9 billion) at urban nonteaching hospitals and an increase of $6.6 billion (95% CI, $5.4 billion to $7.9 billion) at urban teaching hospitals (Figure 1; eTable 1 in Supplement 1). During this period, nonbirth hospitalization costs increased from $23.7 billion to $30.8 billion. Of these, costs decreased by $500.3 million (95% CI, −$613.5 million to −$387.0 million) at rural hospitals and by $2.4 billion (95% CI, −$2.8 billion to −$2.0 billion) at urban nonteaching hospitals. Concurrently, total costs increased by approximately $3.0 billion at urban teaching hospitals and by $6.9 billion at FCHs. In 2019, 56.3% (95% CI, 50.3% to 62.4%) of all nonbirth hospitalizations costs were incurred at FCHs.

The fraction of hospitalization costs covered by Medicaid varied across hospital types (eTable 2 and eTable 3 in Supplement 1). For birth hospitalizations, the proportion covered by Medicaid ranged from 43.1% (95% CI, 40.9%-45.3%) at urban nonteaching hospitals to 52.6% (95% CI, 51.1%-54.2%) at rural hospitals in 2019. For nonbirth hospitalizations, Medicaid coverage ranged from 54.0% (95% CI, 50.3%-57.7%) of hospitalizations at FCHs to 61.6% (95% CI, 59.9%-63.3%) at rural hospitals. Rural hospitals provided care to the largest proportion of children living in the lowest quartile of community median income, including 51.5% (95% CI, 48.8%-54.3%) of birth and 52.9% (95% CI, 48.8%-57.0%) of nonbirth hospitalizations in 2019.

The age distribution for nonbirth hospitalizations also varied across hospital types (eTable 4 in Supplement 1). In 2019, hospitalizations among adolescents aged 15 to 17 years comprised a larger fraction of inpatient stays at rural hospitals (25.5%; 95% CI, 23.7%-27.3%), urban nonteaching hospitals (27.5%; 95% CI, 25.1%-29.9%), and urban teaching hospitals (21.1%; 95% CI, 20.3%-22.0%) than FCHs (13.8%; 95% CI, 13.1%-14.5%). Across hospital types, the proportion of hospitalizations among infants and children younger than 5 years ranged from 46.1% (95% CI, 42.7%-49.6%) at urban nonteaching hospitals to 50.8% (95% CI, 49.2%-52.4%) at FCHs.

### Changes in Clinical Complexity Over Time

**Figure 2.**
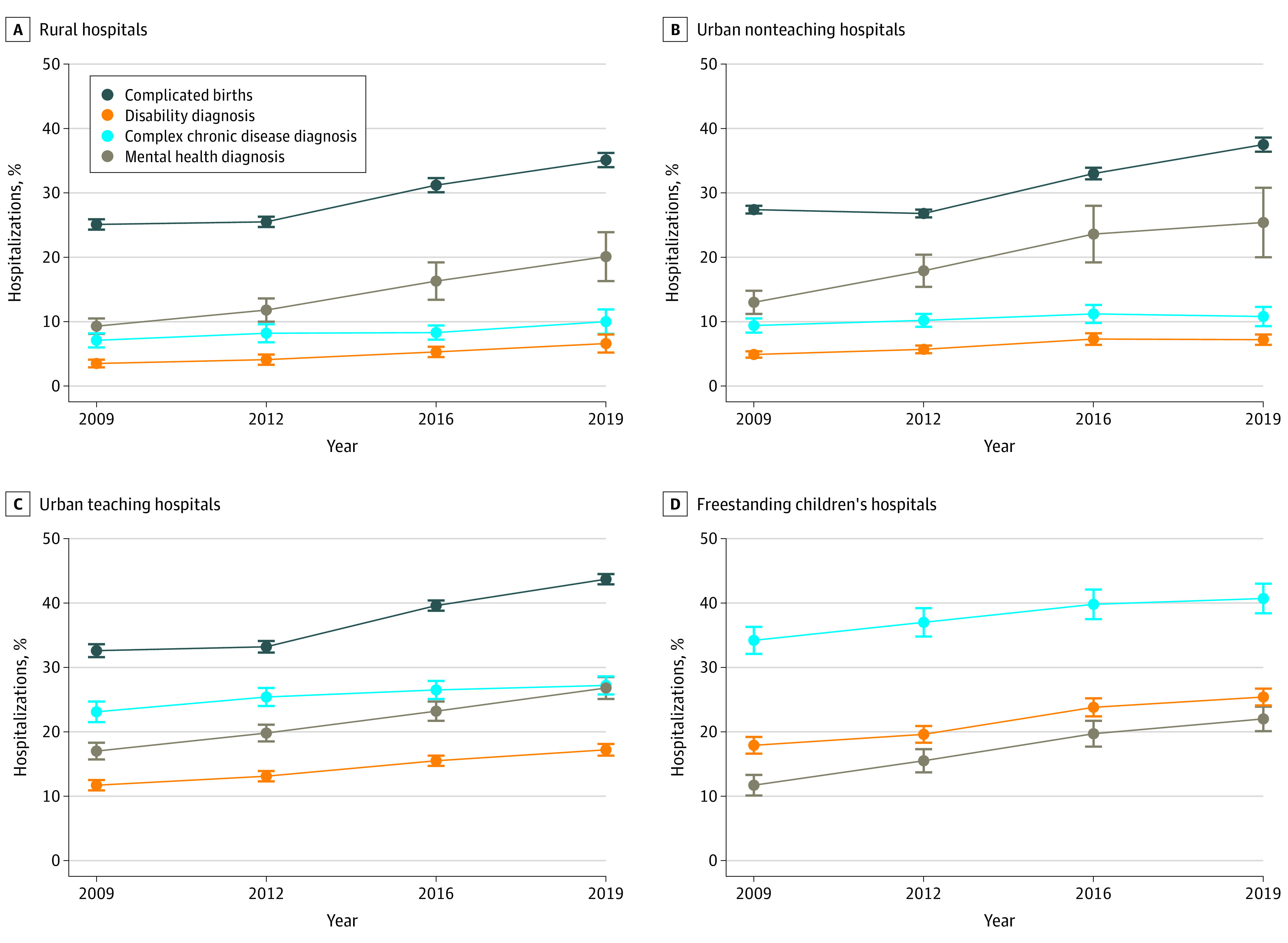
Complicated Birth Hospitalizations and Nonbirth Hospitalizations with Complex Chronic Diseases, Mental Health Diagnoses, and Disability Diagnoses, 2009-2019 Data show weighted national estimates. Error bars denote 95% CIs. The hospital type was missing for 238 818 hospitalizations (3.7%) in 2009.

From 2009 to 2019, there were significant monotonic increases at all hospital types in the proportion of complicated birth hospitalizations, and in nonbirth hospitalizations with complex chronic disease, disability, and mental health diagnoses (Figure 2; eTable 5 in Supplement 1). In 2019, the proportion of complicated birth hospitalizations ranged from 35.1% (95% CI, 34.0%-36.2%) at rural hospitals to 56.1% (95% CI, 51.9%-60.2%) at FCHs, reflecting a relative increase of 42.8% (95% CI, 39.1%-46.5%) overall since 2009. In 2019, the proportion of nonbirth hospitalizations with a complex chronic disease diagnosis varied 4-fold across hospital types, ranging from 10.0% (95% CI, 8.1%-11.9%) at rural hospitals to 40.7% (95% CI, 38.4%-43.0%) at FCHs, with a relative increase of 45.5% (95% CI, 34.6%-56.4%) overall. Approximately 25% of nonbirth hospitalizations had a mental health diagnosis in 2019, reflecting an overall increase of 78.0% (95% CI, 61.6%-94.4%) from 2009 to 2019. The largest increase in mental health hospitalizations occurred at rural hospitals, where they increased by 117.1% (95% CI, 27.5%-206.6%). In 2019, 19.0% (95% CI, 18.2%-19.9%) of hospitalizations were experienced by children with disability diagnoses, ranging from 6.6% (95% CI, 5.2%-8.0%) of hospitalizations at rural hospitals to 25.4% (95% CI, 24.1%-26.8%) at FCHs and representing an overall relative increase of 78.1% (95% CI, 63.1%-93.2%) since 2009.

### Changes in LOS, Costs, and Interfacility Transfers

In all years, geometric mean LOS was longest at FCHs (Table 2). From 2009 to 2019, changes in the geometric mean LOS were not statistically significant except at FCHs, where mean LOS increased by 10% (rate ratio, 1.10; 95% CI, 1.02-1.18). In all years, geometric mean cost per hospitalization varied 6- to 7-fold across hospital types; in 2019, costs ranged from $1644 (95% CI, $1579-$1711) at rural hospitals to $11 922 (95% CI, $10 827-$13 128) at FCHs. During this period, the largest increase in geometric mean cost was at FCHs ($2477; 95% CI, $1120-$3899).

**Table 2.** Length of Stay, Hospitalization Costs, and Interfacility Transfer Rates by Hospital Type, Weighted National Estimates, 2009-2019

Patterns of care	2009 (n = 6 187 040)^a^	2012 (n = 5 857 430)	2016 (n = 5 602 612)	2019 (n = 5 297 882)	Outcome in 2019 vs 2009
Length of stay, geometric mean (95% CI), d	2.4 (2.4 to 2.4)	2.4 (2.4 to 2.4)	2.4 (2.4 to 2.5)	2.4 (2.4 to 2.5)	1.10 (1.06 to 1.14)^b^
Rural hospitals	1.9 (1.9 to 2.0)	1.9 (1.9 to 2.0)	1.9 (1.9 to 1.9)	1.9 (1.9 to 1.9)	0.99 (0.93 to 1.05)^b^
Urban nonteaching hospitals	2.2 (2.2 to 2.2)	2.2 (2.2 to 2.2)	2.2 (2.1 to 2.2)	2.1 (2.1 to 2.1)	0.94 (0.90 to 0.99)^b^
Urban teaching hospitals	2.6 (2.5 to 2.6)	2.6 (2.5 to 2.6)	2.5 (2.5 to 2.5)	2.5 (2.4 to 2.5)	0.97 (0.94 to 1.01)^b^
FCHs	3.1 (2.9 to 3.2)	3.1 (3.0 to 3.2)	3.2 (3.1 to 3.4)	3.2 (3.1 to 3.4)	1.10 (1.02 to 1.18)^b^
Cost, geometric mean (95% CI), $US	2003 (1922 to 2088)	2166 (2073 to 2263)	2328 (2220 to 2441)	2539 (2420 to 2664)	536 (394 to 681)^c^
Rural hospitals	1425 (1385 to 1466)	1456 (1398 to 1516)	1522 (1473 to 1573)	1644 (1579 to 1711)	291 (142 to 297)^c^
Urban nonteaching hospitals	1394 (1349 to 1441)	1442 (1398 to 1487)	1411 (1361 to 1464)	1496 (1428 to 1566)	101 (19 to 185)^c^
Urban teaching hospitals	2230 (2108 to 2358)	2242 (2124 to 2367)	2204 (2102 to 2311)	2290 (2183 to 2402)	60 (−102 to 230)^c^
FCHs	9444 (8694 to 10 259)	9903 (9040 to 10 848)	11 286 (10 251 to 12 425)	11 922 (10 827 to 13 128)	2477 (1120 to 3899)^c^
Transfer from another acute care hospital, No. (%) [95% CI]^d^	210 938 (9.0) [8.3 to 9.8]	248 870 (11.7) [10.9 to 12.6]	265 566 (14.5) [13.5 to 15.5]	317 257 (18.3) [17.0 to 19.7]	2.26 (1.97 to 2.58)^e^
Rural hospitals	4246 (1.9) [1.3 to 2.4]	3810 (2.4) [1.5 to 3.4]	3447 (4.3) [2.6 to 5.9]	3574 (5.7) [3.7 to 7.7]	3.20 (1.95 to 5.27)^e^
Urban nonteaching hospitals	28 197 (4.9) [3.9 to 5.8]	23 141 (6.1) [5.0 to 7.1]	13 216 (7.5) [5.7 to 9.3]	12 315 (13.4) [10.2 to 16.6]	3.03 (2.13 to 4.30)^e^
Urban teaching hospitals	109 980 (11.3) [10.1 to 12.5]	137 618 (13.5) [12.3 to 14.7]	155 702 (15.5) [14.4 to 16.7]	179 954 (17.9) [16.6 to 19.3]	1.71 (1.47 to 1.99)^e^
FCHs	68 515 (12.4) [10.0 to 14.9]	84 301 (14.9) [12.7 to 17.1]	93 200 (16.3) [14.2 to 18.3]	121 413 (21.2) [17.8 to 24.6]	1.90 (1.40 to 2.56)^e^
Transfer to short-term hospital, No. (%) [95% CI]	111 485 (1.8) [1.7 to 1.9]	103 119 (1.8) [1.7 to 1.9]	95 781 (1.7) [1.6 to 1.8]	94 669 (1.8) [1.7 to 1.9]	0.99 (0.92 to 1.07)^e^
Rural hospitals	22 351 (3.2) [3.1 to 3.3]	19 896 (3.3) [3.2 to 3.4]	16 251 (3.6) [3.4 to 3.7]	15 189 (3.8) [3.7 to 4.0]	1.22 (1.15 to 1.29)^e^
Urban nonteaching hospitals	42 632 (1.8) [1.7 to 2.0]	34 560 (1.9) [1.8 to 2.0]	21 952 (2.0) [1.8 to 2.1]	15 828 (2.2) [2.0 to 2.4]	1.21 (1.09 to 1.35)^e^
Urban teaching hospitals	38 893 (1.5) [1.3 to 1.6]	41 933 (1.5) [1.3 to 1.6]	50 927 (1.5) [1.3 to 1.6]	57 161 (1.6) [1.5 to 1.7]	1.08 (0.94 to 1.23)^e^
FCHs	7609 (1.4) [1.0 to 1.7]	6731 (1.2) [0.9 to 1.5]	6651 (1.1) [0.8 to 1.4]	6491 (1.1) [0.8 to 1.3]	0.79 (0.57 to 1.11)^e^

Abbreviation: FCH, freestanding children’s hospital.

^a^ Hospital type (and all hospital characteristics) was missing for 238 818 hospitalizations (3.7%) in 2009.

^b^ Data are rate ratio (95% CI).

^c^ Data are geometric mean difference (95% CI).

^d^ Transfer from another hospital limited to nonbirth hospitalizations, as birth hospitalizations by definition represent in-hospital births.

^e^ Data are odds ratio (95% CI).

The proportion of hospitalizations that followed transfers from another acute care hospital increased significantly at all hospital types from 2009 to 2019. Although rural hospitals received the fewest interfacility transfers, they experienced the largest magnitude of change, with 3.2 times the odds of incoming transfer (odds ratio, 3.20; 95% CI, 1.95-5.27) in 2019 compared with 2009. In contrast, the odds of transfer to another short-term hospital increased modestly from 2009 to 2019 at rural and urban nonteaching hospitals and was not significantly different across years at urban teaching and FCHs.

### Changes in Hospital Pediatric Volume Over Time

The proportion of hospitals in the KID with birth hospitalizations was not significantly different in 2019 vs 2009, but the absolute number decreased from 2784 to 2666, despite a larger number of states represented in the 2019 KID (Table 3). Median birth hospitalization volumes per hospital remained stable overall. The largest decrease from 2009 to 2019 was observed at urban nonteaching hospitals, where the number of hospitals with births decreased from 1149 (65.6%) to 615 (54.9%), and median (IQR) hospital volumes decreased from 1074 (561-1885) to 693 (374-1320) hospitalizations.

**Table 3.** Hospitals in Kids’ Inpatient Database with Birth and Nonbirth Pediatric Hospitalizations and Estimated Per-Hospital Pediatric Volumes, 2009-2019

Characteristics	2009	2012	2016	2019	Change from 2009 to 2019
Hospitals with birth hospitalizations					
Hospitals with births, No./total No. (%)^a^	2784/4121 (67.6)	2760/4179 (66.0)	2747/4200 (65.4)	2666/3988 (66.9)	−1.0 (−4.1 to 2.0)^b^
Hospital volume, median (IQR)	781 (312 to 1811)	780 (311 to 1824)	775 (314 to 1814)	746 (291 to 1771)	−35 (−21 to −40)^c^
Rural hospitals					
Hospitals with births, No./total No. (%)^a^	1011/1589 (63.6)	992/1576 (62.9)	932/1540 (60.5)	890/1401 (63.5)	−0.2 (−5.6 to 5.3)^b^
Hospital volume, median (IQR)	290 (123 to 511)	290 (132 to 499)	272 (128 to 474)	259 (115 to 453)	−31 (−8 to −58)^c^
Urban nonteaching hospitals					
Hospitals with births, No./total No. (%)^a^	1149/1751 (65.6)	1080/1726 (62.6)	824/1404 (58.7)	615/1120 (54.9)	−16.3 (−21.6 to −11.0)^b^
Hospital volume, median (IQR)	1074 (561 to 1885)	983 (519 to 1784)	818 (443 to 1474)	693 (374 to 1320)	−381 (−187 to −565)^c^
Urban teaching hospitals					
Hospitals with births, No./total No. (%)^a^	552/657 (84.0)	678/807 (84.0)	973/1183 (82.2)	1144/1402 (81.6)	−2.9 (−6.9 to 1.2)^b^
Hospital volume, median (IQR)	2353 (1458 to 3564)	2253 (1366 to 3506)	1996 (1125 to 3152)	1741 (944 to 2881)	−612 (−514 to −683)^c^
Freestanding children’s hospitals					
Hospitals with births, No./total No. (%)^a^	8/54 (14.8)	10/70 (14.3)	18/73 (24.7)	17/75 (22.7)	53.0 (−63.9 to 169.9)^b^
Hospital volume, median (IQR)	119 (6 to 2403)	65 (5 to 315)	10 (1 to 571)	429 (5 to 3326)	310 (−1 to 923)^c^
Hospitals with nonbirth pediatric hospitalizations					
Hospitals with pediatric stays, No./total No. (%)^a^	3985/4121 (96.7)	3940/4179 (94.2)	3808/4200 (90.7)	3507/3988 (87.9)	−9.1 (−10.2 to −7.9)^b^
Hospital volume, median (IQR)	99 (24 to 364)	69 (15 to 271)	44 (10 to 189)	38 (9 to 176)	−61 (−15 to −188)^c^
Rural hospitals					
Hospitals with pediatric stays, No./total No. (%)^a^	1563/1589 (98.4)	1525/1576 (96.8)	1425/1540 (92.5)	1256/1401 (89.7)	−8.9 (−10.6 to −7.1)^b^
Hospital volume, median (IQR)	50 (15 to 143)	34 (10 to 99)	19 (5 to 55)	17 (5 to 44)	−33 (−10 to −99)^c^
Urban nonteaching hospitals					
Hospitals with pediatric stays, No./total No. (%)^a^	1648/1751 (94.1)	1555/1726 (90.1)	1165/1404 (83.0)	843/1120 (75.3)	−20.0 (−22.9 to −17.2)^b^
Hospital volume, median (IQR)	103 (23 to 361)	65 (13 to 243)	34 (8 to 126)	25 (6 to 75)	−78 (−17 to −286)^c^
Urban teaching hospitals					
Hospitals with pediatric stays, No./total No. (%)^a^	652/657 (99.2)	790/807 (97.9)	1145/1183 (96.8)	1333/1402 (95.1)	−4.2 (−5.5 to −2.9)^b^
Hospital volume, median (IQR) (IQR)	611 (193 to 1597)	438 (118 to 1351)	210 (50 to 794)	149 (34 to 645)	−462 (−159 to −952)^c^
Freestanding children’s hospitals					
Hospitals with pediatric stays, No./total No. (%)^a^	54/54 (100)	70/70 (100)	73/73 (100)	75/75 (100)	Not applicable
Hospital volume, median (IQR)	7694 (2170 to 11 783)	6790 (415 to 11 215)	6682 (514 to 11 243)	6059 (1036 to 11 308)	−1635 (−1134 to −475)^c^

^a^ Denominator reflects all hospitals included in the Kids’ Inpatient Database each year. Hospital type and all hospital characteristics are missing for 238 818 hospitalizations (3.7%) in 2009.

^b^ Data are percentage relative change (95% CI).

^c^ Data are differences in medians (IQRs). Note that the difference in medians reflects differences in the distribution across years, not the distribution of the changes.

Compared with birth hospitalization care, a larger number of hospitals represented in the KID provided nonbirth pediatric care. The number and proportion of hospitals with nonbirth pediatric hospitalizations decreased from 3985 hospitals (96.7%) in 2009 to 3507 hospitals (87.9%) in 2019. These changes were accompanied by year-over-year decreases in median hospital volumes at all 4 hospital types. The most substantial decrease was observed at urban nonteaching hospitals, where the number and proportion of hospitals admitting children decreased from 1648 (94.1%) in 2009 to 843 (75.3%) in 2019, and the median (IQR) volume decreased from 103 (23-361) to 25 (6-75) hospitalizations. The number of rural hospitals with nonbirth hospitalizations decreased from 1563 (98.4%) in 2009 to 1256 (89.7%) in 2019, with annual median (IQR) volumes decreasing from 50 (15-143) to 17 (5-44) hospitalizations.

## Discussion

This retrospective, cross-sectional analysis of nationally representative data found significant decreases in both birth and nonbirth pediatric hospitalizations from 2009 to 2019, with shifts in where care was provided. In urban communities, birth and nonbirth hospitalizations shifted from nonteaching to teaching hospitals, while the number of hospitalizations at FCHs remained relatively stable. In rural communities, birth hospitalizations decreased by approximately 25%, while nonbirth hospitalizations decreased 4-fold. These findings align with national birth statistics and obstetrics-focused research.^18,19,37^ Throughout this period, an increasing proportion of hospitalizations in all settings were experienced by children with disabilities, mental health diagnoses, and complex chronic diseases. In 2019, approximately two-thirds of hospitals in the data set provided birth hospitalization care, and nonbirth pediatric hospitalizations occurred in more than 3500 hospitals.

These findings build on a prior analysis^6^ of AHA data that found a substantial decrease from 2008 to 2018 in the number of US hospitals with pediatric units, from 1753 to 1418 hospitals. Using data from a similar period, the present study illustrates that pediatric hospitalizations occurred at a much larger number of hospitals, including 1256 rural hospitals, 1333 urban teaching hospitals, and 843 urban nonteaching hospitals, in addition to FCHs. Prior single-state analyses^38^ have shown that even following pediatric unit closures, almost one-quarter of hospitals continue to admit children at volumes similar to those experienced before unit closure. This national study supports these findings, because the number of hospitals admitting children exceeded the number of hospitals reported to have pediatric units by a factor of 2.5. More than two-thirds of nonbirth hospitalizations at rural and urban nonteaching hospitals were for children younger than 15 years, and almost 50% were for children younger than 5 years, suggesting that these trends are not associated with hospital admissions among older adolescents.

Hospitalization of children at hospitals without pediatric units may reduce travel times and health care costs (both direct and indirect) while helping to maintain hospitals’ pediatric competence. Although the association of a hospital’s volume of common pediatric nonsurgical conditions with health outcomes has received little study, research has demonstrated higher mortality in children with critical illnesses who present to emergency departments with limited pediatric readiness.^39,40^ Taken together, these findings speak to the need for clinical and policy initiatives to support care for children at hospitals without pediatric units, recognizing that children will continue to present to these facilities for care. Telehealth programs, interfacility transfer guidelines, and educational collaboratives between general hospitals and children’s hospitals may support both health care quality and access to care.^41,42,43^

Across all hospital types, Medicaid was a predominant payer, covering 43.1% to 52.6% of birth hospitalizations and 54.0% to 61.6% of nonbirth hospitalizations. Rural hospitals had the largest proportion of hospitalizations covered by Medicaid, and more than one-half of rural hospitalizations were experienced by children living in communities with the lowest median incomes. These findings are consistent with prior obstetrics-focused research.^44,45,46^ Poverty is a well-established risk factor for pediatric hospitalization; rural-residing children also experience greater unmet health care needs, increased rates of several chronic diseases, and higher child mortality than their urban-residing peers.^47,48,49,50,51,52^ However, low population densities, health professional shortages, substantial hospital fixed costs, and low Medicaid reimbursement rates may contribute to rural hospital closures and loss of dedicated pediatric services at the hospitals that remain open. The 2021 Consolidated Appropriations Act established the Rural Emergency Hospital designation, providing eligible rural hospitals with additional annual facility payments and increased reimbursement for some services in exchange for inpatient unit closure.^53^ Although both opportunities and challenges of this program have been acknowledged, its potential effects on pediatric health care access and quality have received little attention.^54^ Recognizing that interfacility transfers are not without risks and can laden families with substantial additional costs,^55,56^ concerted efforts are needed to ensure that decreases in rural hospital pediatric care do not serve to widen rural-urban disparities.

### Limitations

This study has limitations that should be acknowledged. The KID does not contain data from all states, and it includes only hospitals that provided care to patients aged 20 years or younger. Therefore, our results may overestimate the proportion of hospitals with pediatric inpatient hospitalizations and underestimate the absolute number of pediatric-serving hospitals. After 2009, KID hospital identifiers were reassigned yearly, so hospitals cannot be linked across years. Hospital identifiers are also encrypted and cannot be linked to external data, such as the AHA annual survey, so it is not possible to differentiate between general hospitals with and without pediatric units. Beginning in 2014, more hospitals were categorized as urban teaching hospitals (vs urban nonteaching hospitals) as more facilities reported approved residency programs in the AHA Annual Survey.^21^ Therefore, we cannot differentiate between care that shifted between hospitals from that provided within the same, recategorized hospital. However, trends both before and after 2014 demonstrate similar patterns of increasing hospitalizations at urban teaching hospitals. Additionally, the KID does not contain an indicator of observation status stays, which vary substantially across hospitals.^57,58,59,60^ Recent analyses have shown increased observation status coding at FCH hospitalizations from 2010 to 2019; less is known about trends in observation status coding for pediatric stays at general hospitals.^60^ Accordingly, the decreasing number of inpatient hospitalizations noted in this study may be influenced by increased observation status coding and exclusion of these stays from the KID. Increases in the proportion of complicated birth hospitalizations and nonbirth hospitalizations with chronic condition diagnoses are marked. Although increases in the proportion of children with medical complexity, disability, and mental health conditions are well established, we cannot ascertain the extent to which our findings were also influenced by changing coding and billing practices over time.

## Conclusions

In this national cross-sectional study of pediatric inpatient hospitalization data, both birth and nonbirth hospitalizations decreased substantially from 2009 to 2019, with rural and urban nonteaching hospitals experiencing the largest decreases. The proportion of hospitalizations for children with complex diagnoses increased concurrently. In 2019, hospitalizations occurred in more than 3500 hospitals, more than twice the number of hospitals reported previously to have dedicated pediatric units. Better characterization of the pediatric capabilities, resources, and outcomes at these hospitals is needed to inform both health policy and clinical decision-making.

## Notes

Supplement 1. **eTable 1.** Birth and Nonbirth Pediatric Hospitalizations and Total Hospitalization Costs at Rural Hospitals, Urban Nonteaching and Teaching Hospitals, and Freestanding Children’s Hospitals, 2009-2019, Weighted National Estimates **eTable 2.** Payer and Community Median Income Distribution by Year and Hospital Type Among Birth Hospitalizations, Weighted National Estimates, 2009-2019^a^ **eTable 3.** Payer and Community Median Income Distribution by Year and Hospital Type Among Nonbirth Pediatric Hospitalizations, Weighted National Estimates, 2009-2019^a^ **eTable 4.** Age Distribution by Year and Hospital Type Among Nonbirth Pediatric Hospitalizations, Weighted National Estimates, 2009-2019 **eTable 5.** Complicated Birth Hospitalizations and Nonbirth Hospitalizations With Complex Chronic Diseases, Mental Health, and Disability Diagnoses, Weighted National Estimates, 2009-2019

Supplement 2.
Data Sharing Statement
