# Supplementary material for: Pediatric Hospitalizations at Rural and Urban Teaching and Nonteaching Hospitals in the US, 2009-2019
Source: JAMA Netw Open. 2023 Sep 1;6(9):e2331807. doi: 10.1001/jamanetworkopen.2023.31807 (PMC10474556; doi:10.1001/jamanetworkopen.2023.31807)
Supplement: Supplement 1. — eTable 1. Birth and Nonbirth Pediatric Hospitalizations and Total Hospitalization Costs at Rural Hospitals, Urban Nonteaching and Teaching Hospitals, and Freestanding Children’s Hospitals, 2009-2019, Weighted National Estimates eTable 2. Payer and Community Median Income Distribution by Year and Hospital Type Among Birth Hospitalizations, Weighted National Estimates, 2009-2019a eTable 3. Payer and Community Median Income Distribution by Year and Hospital Type Among Nonbirth Pediatric Hospitalizations, Weighted National Estimates, 2009-2019a eTable 4. Age Distribution by Year and Hospital Type Among Nonbirth Pediatric Hospitalizations, Weighted National Estimates, 2009-2019 eTable 5. Complicated Birth Hospitalizations and Nonbirth Hospitalizations With Complex Chronic Diseases, Mental Health, and Disability Diagnoses, Weighted National Estimates, 2009-2019 [file jamanetwopen-e2331807-s001.pdf]

Supplemental Online Content

Leyenaar JK, Freyleue S, Arakelyan M, Goodman D, O'Malley AJ. Pediatric hospitalizations at rural and urban teaching and nonteaching hospitals in the US, 2009-2019. *JAMA Netw Open*. 2023;6(9):e2331807. doi:10.1001/jamanetworkopen.2023.31807

eTable 1. Birth and Nonbirth Pediatric Hospitalizations and Total Hospitalization Costs at Rural Hospitals, Urban Nonteaching and Teaching Hospitals, and Freestanding Children's Hospitals, 2009-2019, Weighted National Estimates

eTable 2. Payer and Community Median Income Distribution by Year and Hospital Type Among Birth Hospitalizations, Weighted National Estimates, 2009-2019[a]

eTable 3. Payer and Community Median Income Distribution by Year and Hospital Type Among Nonbirth Pediatric Hospitalizations, Weighted National Estimates, 2009-2019[a]

eTable 4. Age Distribution by Year and Hospital Type Among Nonbirth Pediatric Hospitalizations, Weighted National Estimates, 2009-2019

eTable 5. Complicated Birth Hospitalizations and Nonbirth Hospitalizations With Complex Chronic Diseases, Mental Health, and Disability Diagnoses, Weighted National Estimates, 2009-2019

This supplemental material has been provided by the authors to give readers additional information about their work.

© 2023 Leyenaar JK et al. *JAMA Network Open.*

eTable 1. Birth and Nonbirth Pediatric Hospitalizations and Total Hospitalization Costs at Rural Hospitals, Urban Nonteaching and Teaching Hospitals, and Freestanding Children's Hospitals, 2009-2019, Weighted National Estimates.

	**2009**[a]<br>N,% of total (95%CI)	**2012**<br>N, % of total (95%CI)	**2016**<br>N, % of total (95%CI)	**2019**<br>N, % of total (95%CI)	**Change, 2009-2019**<br>N (95% CI)
**Birth hospitalizations**	**3,856,059**	**3,736,337**	**3,770,047**	**3,567,900**	**-288,159<br>(-479,497; -96,821)**
Rural hospitals	474,038<br>12.3 (11.6-13.0)	447,988,<br>12.0 (11.3-12.6)	372,614,<br>9.9 (9.3-10.4)	332,835,<br>9.3 (8.8-9.9)	-141,203<br>(-171,093; -111,313)
Urban non-teaching hospitals	1,723,546<br>44.7 (42.9-46.5)	1,429,665,<br>38.3 (36.7-39.8)	929,780,<br>24.7 (23.3-26.0)	617,957,<br>17.3 (16.2-18.5)	-1,105,589<br>(-1,202,886; -1,008,292)
Urban teaching hospitals	1,647,202<br>42.7 (40.8-44.6)	1,845,633,<br>49.4 (47.7-51.1)	2,447,520,<br>64.9 (63.4-66.4)	2,586,803,<br>72.5 (71.1-73.9)	+939,601<br>(779,872; 1,099,330)
Freestanding children's hospitals	11,273<br>0.3 (0.0-0.7)	13,051,<br>0.3 (0.0-0.8)	20,133,<br>0.5 (0.0-1.1)	30,305,<br>0.8 (0.2-1.5)	+19,032<br>(-8,106; 46,170)
**Birth hospitalization costs, Millions of USD**	**11,600.6**	**13,401.5**	**15,643.0**	**16,699.7**	Millions of USD<br>**+5,099.1<br>(3,673.1; 6,525.2)**
Rural hospitals	636.8<br>5.5 (4.9-6.1)	703.8<br>5.3 (4.6-5.9)	637.6<br>4.1 (3.7-4.5)	650.6<br>3.9 (3.4-4.4)	+13.8<br>(-78.7; 106.4)
Urban-nonteaching hospitals	3,939.5<br>34.0 (31.9-36.0)	3,534.1<br>26.4 (24.7-28.1)	2,268.0<br>14.5 (13.0-16.0)	1,637.1<br>9.8 (8.6-11.1)	-2,302.5<br>(-2,716.3; -1,888.7)
Urban-Teaching hospitals	6,896.9<br>59.5 (56.8-62.1)	8,852.5<br>66.1 (63.6 68.5)	11,932.2<br>76.3 (73.7-78.8)	13,551.7<br>81.1 (78.5-83.8)	+6,654.8<br>(5,416.8; 7,892.9)
Freestanding children's hospitals	127.4<br>1.1 (0.0-2.4)	311.1<br>2.3 (0.3-4.3)	805.2<br>5.1 (1.7-8.6)	860.4<br>5.2 (2.2-8.1)	+733.0<br>(166.4; 1,299.5)
**Non-birth hospitalizations**	**2,330,980**	**2,121,093**	**1,832,564**	**1,729,982**	**-600,998<br>(-811,907; -390,089)**
Rural hospitals	229,263<br>9.8 (9.0-10.7)	156,428<br>7.4 (6.6-8.1)	80,705<br>4.4 (3.9-4.9)	62,729<br>3.6 (3.2-4.1)	-166,534<br>(-182,487; -150,581)
Urban non-teaching hospitals	581,320<br>24.9 (22.7-27.2)	381,642<br>18.0 (16.2-19.8)	177,222<br>9.7 (8.3-11.0)	92,118<br>5.3 (4.5-6.1)	-489,202<br>(-543,358; -435,046)
Urban teaching hospitals	969,631<br>41.6 (38.4-44.8)	1,017,888<br>48.0 (44.0.3-51.6)	1,001,976<br>54.7 (50.4-58.9)	1,002,652<br>58.0 (53.5-62.4)	+33,021<br>(-98,395; 164,437)
Freestanding children's hospitals	550,766<br>23.6 (20.0-27.3)	565,135<br>26.6 (22.4-30.9)	572,661<br>31.2 (26.5-36.0)	572,483<br>33.1 (28.4-37.8)	+21,717<br>(-133,283; 176,717)
**Non-birth hospitalization costs, Millions of USD**	**23,739.0**	**26,948.3**	**28,897.6**	**30,782.6**	Millions of USD<br>**+7,043.6<br>(1,939; 12,148.2)**
Rural hospitals	865.1<br>3.6 (3.1-4.2)	747.9<br>2.8 (2.0-3.6)	397.8<br>1.4 (1.1-1.6)	364.8<br>1.2 (0.9-1.5)	-500.3<br>(-613.5; -387.0)
Urban non-teaching hospitals	3,049.5<br>12.8 (11.1-14.6)	2,097.9<br>7.8 (6.7-8.9)	1,020.8<br>3.5 (2.9-4.2)	614.5<br>2.0 (1.5-2.5)	-2,435.0<br>(-2,828.0; -2,042.0)
Urban teaching hospitals	9,424.7<br>39.7 (35.9-43.5)	10,878.1<br>40.4 (36.5-44.2)	11,700.0<br>40.5 (36.5-44.5)	12,461.6<br>40.5 (36.5-44.5)	+3,036.9<br>(819.2; 5,254.7)
Freestanding children's hospitals	10,399.7<br>43.8 (38.3-49.4)	13,224.4<br>49.1 (43.4-54.8)	15,779.1<br>54.6 (48.5-60.7)	17,341.6<br>56.3 (50.3-62.4)	+6,941.9<br>(2,362.5; 11,521.4)

[a] Hospital type (and all hospital characteristics) missing for 238,818 (3.7%) of hospitalizations in 2009

© 2023 Leyenaar JK et al. *JAMA Network Open.*

eTable 2. Payer and Community Median Income Distribution by Year and Hospital Type Among Birth Hospitalizations, Weighted National Estimates, 2009-2019[a]

	2009[d]		2012		2016		2019	
**Rural Hospitals**	**N**	**%, 95% CI**	**N**	**%, 95% CI**	**N**	**%, 95% CI**	**N**	**%, 95% CI**
**Primary Payer**	**472,941**		**445,664**		**371,700**		**332,138**	
Medicaid	261,588	55.3(53.8-56.8)	242,757	54.5(52.9-56.1)	195,225	52.5(50.9-54.1)	174,727	52.6(51.1-54.2)
Medicare	1,759	0.4(0-0.7)	2,453	0.6(0.1-1.0)	1,518	0.4(0.2-0.7)	675	0.2(0.1-0.3)
Private insurance	166,703	35.2(33.9-36.6)	158,423	35.5(34.2-36.9)	139,223	37.5(36.0-39.0)	120,435	36.3(34.9-37.7)
Other	18,627	3.9(3.2-4.7)	20,581	4.6(3.7-5.6)	12,869	3.5(2.8-4.2)	9,085	2.7(2.2-3.3)
Self-pay	24,264	5.1(4.4-5.9)	21,450	4.8(4.1-5.5)	22,865	6.2(5.5-6.9)	27,216	8.2(7.2-9.2)
**Median Income at ZIP code**[bc]	**459,509**		**435,237**		**364,076**		**327,040**	
Quartile 1	228,758	49.8(47.0-52.6)	224,524	51.6(48.9-54.3)	167,128	45.9(43.3-48.5)	168,490	51.5(48.8-54.3)
Quartile 2	166,334	36.2(34.0-38.4)	150,195	34.5(32.3-36.7)	137,993	37.9(35.7-40.1)	110,761	33.9(31.6-36.1)
Quartile 3	53,868	11.7(10.1-13.3)	48,758	11.2(9.7-12.7)	50,653	13.9(12.3-15.5)	40,424	12.4(10.7-14.0)
Quartile 4	10,549	2.3(1.5-3.1)	11,760	2.7(1.8-3.6)	8,302	2.3(1.4-3.1)	7,365	2.3(1.4-3.1)
**Urban Non-Teaching Hospitals**								
**Primary Payer**	**1,722,020**		**1,426,877**		**928,817**		**617,617**	
Medicaid	744,126	43.2(41.5-44.9)	634,556	44.5(42.8-46.1)	427,400	46.0(44.1-47.9)	266,085	43.1(40.9-45.3)
Medicare	1,899	0.1(0.1-0.2)	5,350	0.4(0.2-0.6)	3,586	0.4(0.2-0.5)	3,172	0.5(0.3-0.7)
Private insurance	862,057	50.1(48.3-51.8)	677,472	47.5(45.8-49.2)	419,143	45.1(43.2-47.1)	291,856	47.3(45.0-49.5)
Other	46,406	2.7(2.4-3.0)	52,447	3.7(3.2-4.2)	28,597	3.1(2.7-3.5)	18,669	3.0(2.6-3.5)
Self-pay	67,532	3.9(3.5-4.4)	57,052	4.0(3.4-4.6)	50,091	5.4(4.6-6.2)	37,835	6.1(5.3-7.0)
**Median Income at ZIP code**[b]	**1,698,580**		**1,411,176**		**920,216**		**611,558**	
Quartile 1	361,424	21.3(19.6-23.0)	310,365	22.0(20.3-23.7)	233,071	25.3(23.4-27.3)	135,450	22.1(19.9-24.4)
Quartile 2	434,685	25.6(24.3-26.9)	340,183	24.1(22.8-25.4)	228,754	24.9(23.5-26.3)	158,856	26.0(24.1-27.9)
Quartile 3	477,750	28.1(26.9-29.4)	393,042	27.9(26.6-29.1)	246,665	26.8(25.4-28.2)	171,808	28.1(26.4-29.8)
Quartile 4	424,721	25.0(23.0-27.0)	367,586	26.0(23.9-28.2)	211,726	23.0(20.7-25.3)	145,444	23.8(20.9-26.7)
**Urban Teaching Hospitals**								
**Primary Payer**	**1,644,144**		**1,840,555**		**2,445,003**		**2,583,107**	
Medicaid	730,221	44.4(41.9-46.9)	844,499	45.9(43.8-47.9)	1,084,288	44.3(42.7-46.0)	1,130,471	43.8(42.3-45.2)
Medicare	1,647	0.1(0.0-0.2)	5,467	0.3(0.2-0.4)	8,644	0.4(0.2-0.5)	5,324	0.2(0.1-0.3)
Private insurance	801,147	48.7(46.1-51.4)	878,556	47.7(45.6-49.8)	1,186,694	48.5(46.9-50.2)	1,253,935	48.5(47-50.1)
Other	36,688	2.2(1.8-2.7)	45,862	2.5(2.1-2.8)	65,743	2.7(2.3-3.0)	66,780	2.6(2.3-2.9)
Self-pay	74,441	4.5(3.8-5.3)	66,171	3.6(3.1-4.1)	99,634	4.1(3.7-4.5)	126,597	4.9(4.4-5.4)

© 2023 Leyenaar JK et al. *JAMA Network Open.*

	2009		2012		2016		2019	
	**N**	**%, 95% CI**	**N**	**%, 95% CI**	**N**	**%, 95% CI**	**N**	**%, 95% CI**
**Median Income at ZIP code** [b]	**1,611,398**		**1,811,661**		**2,426,960**		**2,568,208**	
Quartile 1	451,010	28(25.8-30.2)	505,675	27.9(26.0-29.8)	657,658	27.1(25.6-28.6)	677,060	26.4(24.9-27.8)
Quartile 2	386,508	24(22.7-25.3)	410,097	22.6(21.5-23.8)	544,524	22.4(21.4-23.4)	588,211	22.9(21.9-23.9)
Quartile 3	402,554	25(23.7-26.2)	459,873	25.4(24.3-26.5)	626,354	25.8(24.9-26.7)	675,376	26.3(25.4-27.2)
Quartile 4	371,326	23(20.8-25.3)	436,016	24.1(22.0-26.1)	598,424	24.7(22.9-26.4)	627,561	24.4(22.8-26.1)
**Freestanding Children's Hospital**								
**Primary Payer**	**11,273**		**13,051**		**20,134**		**30,305**	
Medicaid/Medicare	5,346	47.4(45.3-49.5)	6,129	47.0(41.1-52.9)	9,707	48.2(33.7-62.7)	13,962	46.1(39.6-52.5)
Private Insurance	5,472	48.5(43.1-54.0)	6,227	47.7(40.9-54.5)	9,056	45.0(35.4-54.6)	14,299	47.2(41.0-53.3)
Other	325	2.9(0.1-5.7)	414	3.2(2.5-3.9)	1,072	5.3(0.1-10.5)	1,052	3.5(1.2-5.7)
Self-pay	130	1.2(0.0-2.4)	281	2.2(0.3-4.0)	299	1.5(0.2-2.8)	992	3.3(1.5-5.0)
**Median Income at ZIP code** [b]	**11,194**		**12,915**		**20,011**		**30,083**	
Quartile 1	2,426	21.7(0.0-46.4)	2,038	15.8(0.0-32.6)	3,455	17.3(7.1-27.4)	5,559	18.5(7.2-29.8)
Quartile 2	1,463	13.1(0.0-27.0)	2,731	21.1(0.0-42.3)	5,560	27.8(6.5-49.1)	6,069	20.2(7.3-33.1)
Quartile 3	2,148	19.2(17.7-20.7)	1,951	15.1(12.1-18.1)	3,678	18.4(13.1-23.7)	7,830	26.0(17.8-34.3)
Quartile 4	5,157	46.1(6.2-85.9)	6,195	48.0(7.2-88.7)	7,318	36.6(5.8-67.4)	10,625	35.3(16.5-54.2)

[a] Bolded numbers show weighted national estimates of non-missing data; variables with ≥3.0% missing data are also indicated with footnotes;

[b] Quartile ranges for median household income at home ZIP Code vary annually (e.g. Quartile 1 encompasses median household incomes of <$40,000 in 2009 and <$48,000 in 2019.) Details available in the HCUP data dictionaries.

[c] n, % of hospitalizations with missing values in 2019 - 14,529 (3.0%)

[d] Hospital type missing for 238,818 (3.7%) of hospitalizations in 2009

© 2023 Leyenaar JK et al. *JAMA Network Open.*

eTable 3. Payer and Community Median Income Distribution by Year and Hospital Type Among Nonbirth Pediatric Hospitalizations, Weighted National Estimates, 2009-2019[a]

	2009[d]		2012		2016		2019	
**Rural Hospitals**	**N**	**%, 95% CI**	**N**	**%, 95% CI**	**N**	**%, 95% CI**	**N**	**%, 95% CI**
**Primary Payer**	**228,576**		**155,380**		**80,252**		**62,488**	
Medicaid	133,437	58.4(56.9-59.9)	91,660	59.0(57.3-60.7)	47,766	59.5(57.4-61.6)	38,517	61.6(59.9-63.3)
Medicare	1,221	0.5(0.0-1.2)	967	0.6(0.2-1.0)	408	0.5(0.2-0.9)	233	0.4(0.1-0.6)
Private insurance	78,140	34.2(32.8-35.6)	52,032	33.5(31.9-35.1)	26,960	33.6(31.4-35.8)	19,684	31.5(29.8-33.2)
Other	8,147	3.6(3.1-4.1)	6,009	3.9(3.1-4.7)	2,613	3.3(2.5-4.0)	1,713	2.7(2.0-3.5)
Self-pay	7,631	3.3(3.1-3.6)	4,712	3.0(2.8-3.3)	2,505	3.1(2.8-3.4)	2,341	3.7(3.4-4.1)
**Median Income at ZIP code** [bc]	**221,172**		**151,517**		**78,490**		**61,211**	
Quartile 1	130,793	59.1(56.3-62.0)	91,451	60.4(57.4-63.3)	40,639	51.8(48.3-55.3)	32,382	52.9(48.8-57.0)
Quartile 2	67,555	30.5(28.4-32.7)	44,598	29.4(27.0-31.8)	26,194	33.4(30.6-36.2)	19,812	32.4(29.3-35.4)
Quartile 3	18,534	8.4(7.1-9.7)	12,693	8.4(7.1-9.7)	9,747	12.4(10.6-14.2)	7,458	12.2(10.1-14.2)
Quartile 4	4,290	1.9(1.3-2.6)	2,775	1.8(1.3-2.3)	1,910	2.4(1.6-3.2)	1,559	2.5(1.6-3.5)
**Urban Non-Teaching Hospitals**								
**Primary Payer**	**580,624**		**380,822**		**177,135**		**92,062**	
Medicaid	291,733	50.2(48.4-52.1)	202,491	53.2(51.3-55.0)	101,111	57.1(54.5-59.7)	52,011	56.5(53.5-59.5)
Medicare	845	0.1(0.1-0.2)	896	0.2(0.1-0.4)	634	0.4(0.1-0.6)	568	0.6(0.2-1.0)
Private insurance	246,417	42.4(40.6-44.3)	149,234	39.2(37.2-41.2)	64,298	36.3(33.4-39.2)	32,556	35.4(32.6-38.1)
Other	21,895	3.8(3.3-4.2)	17,330	4.6(3.9-5.2)	5,990	3.4(2.7-4.1)	3,346	3.6(3.0-4.2)
Self-pay	19,734	3.4(3.1-3.7)	10,871	2.9(2.6-3.1)	5,102	2.9(2.5-3.2)	3,581	3.9(3.2-4.6)
**Median Income at ZIP code** [b]	**570,325**		**375,728**		**174,933**		**91,047**	
Quartile 1	159,787	28.0(25.3-30.7)	112,569	30.0(26.8-33.1)	58,468	33.4(29.5-37.4)	27,684	30.4(26.2-34.6)
Quartile 2	153,614	26.9(25.5-28.4)	96,461	25.7(24.0-27.3)	44,530	25.5(23.5-27.4)	24,903	27.4(24.7-30.0)
Quartile 3	143,315	25.1(23.5-26.7)	89,690	23.9(22.3-25.4)	40,904	23.4(21.4-25.3)	22,460	24.7(22.3-27.0)
Quartile 4	113,609	19.9(17.8-22.0)	77,008	20.5(18.0-23.0)	31,031	17.7(15.0-20.5)	16,000	17.6(13.9-21.3)
**Urban Teaching Hospitals**								
**Primary Payer**	**968,324**		**1,015,210**		**1,001,060**		**1,001,448**	
Medicaid	474,462	49.0(47.2-50.8)	538,405	53.0(51.3-54.7)	554,190	55.4(53.9-56.8)	548,851	54.8(53.2-56.4)
Medicare	2,878	0.3(0.2-0.4)	4,256	0.4(0.2-0.6)	3,992	0.4(0.2-0.6)	2,807	0.3(0.2-0.4)
Private insurance	418,095	43.2(41.2-45.1)	402,345	39.6(37.8-41.5)	381,247	38.1(36.6-39.5)	384,579	38.4(36.8-40.0)
Other	35,420	3.7(3.1-4.2)	44,385	4.4(3.4-5.3)	38,126	3.8(3.2-4.4)	35,924	3.6(3.1-4.1)
Self-pay	37,469	3.9(3.2-4.6)	25,819	2.5(2.3-2.8)	23,505	2.3(2.2-2.5)	29,287	2.9(2.7-3.2)

© 2023 Leyenaar JK et al. *JAMA Network Open.*

	2009		2012		2016		2019	
	**N**	**%, 95% CI**	**N**	**%, 95% CI**	**N**	**%, 95% CI**	**N**	**%, 95% CI**
**Median Income at ZIP code** [b]	**940,963**		**990,372**		**989,088**		**991,987**	
Quartile 1	296,826	31.5(29.0-34.0)	313,717	31.7(29.5-33.9)	323,690	32.7(30.6-34.8)	321,206	32.4(30.2-34.6)
Quartile 2	236,832	25.2(23.8-26.5)	243,771	24.6(23.3-25.9)	240,014	24.3(23.1-25.4)	246,543	24.9(23.6-26.1)
Quartile 3	214,489	22.8(21.6-24.0)	232,093	23.4(22.4-24.5)	232,217	23.5(22.5-24.5)	237,278	23.9(22.9-24.9)
Quartile 4	192,816	20.5(18.2-22.8)	200,791	20.3(18.2-22.4)	193,167	19.5(17.5-21.5)	186,960	18.8(16.9-20.8)
**Freestanding Children's Hospital**								
**Primary Payer**	**549,917**		**564,916**		**571,131**		**571,421**	
Medicare/Medicaid	265,747	48.3(44.0-52.6)	288,612	51.1(47.2-55.0)	297,742	52.1(47.8-56.5)	308,675	54.0(50.3-57.7)
Private insurance	241,883	44.0(39.8-48.2)	228,876	40.5(37.2-43.9)	232,275	40.7(36.9-44.4)	221,761	38.8(35.9-41.7)
Other	28,478	5.2(3.0-7.4)	30,031	5.3(3.0-7.6)	26,284	4.6(2.3-6.9)	24,831	4.3(2.1-6.5)
Self-pay	13,809	2.5(1.7-3.4)	17,397	3.1(1.8-4.3)	14,830	2.6(1.9-3.3)	16,154	2.8(2.2-3.5)
**Median Income at ZIP code** [b]	**542,291**		**556,698**		**563,243**		**564,507**	
Quartile 1	159,109	29.3(24.6-34.1)	167,037	30.0(25.4-34.6)	161,618	28.7(24.4-33.0)	159,259	28.2(23.9-32.5)
Quartile 2	128,552	23.7(21.5-26.0)	127,719	22.9(20.9-25.0)	132,249	23.5(21.1-25.9)	129,419	22.9(20.6-25.2)
Quartile 3	133,747	24.7(22.0-27.3)	137,334	24.7(22.2-27.2)	138,767	24.6(22.6-26.6)	144,676	25.6(23.4-27.9)
Quartile 4	120,883	22.3(18.1-26.4)	124,608	22.4(18.5-26.3)	130,609	23.2(19.1-27.3)	131,153	23.2(19.3-27.1)

[a] Bolded numbers show weighted national estimates of non-missing data; variables with >3.0% missing data are also indicated with footnotes

[b] Quartile ranges for median household income at home ZIP Code vary annually (e.g. Quartile 1 encompasses median household incomes of <$40,000 in 2009 and <$48,000 in 2019.) Details available in the HCUP data dictionaries.

[c] N, % of hospitalizations with missing values: 2009: 8,091 (3.5%) 2012: 4,911 (3.1%)

[d] Hospital type missing for 238,818 (3.7%) of hospitalizations in 2009

© 2023 Leyenaar JK et al. *JAMA Network Open.*

eTable 4. Age Distribution by Year and Hospital Type Among Nonbirth Pediatric Hospitalizations, Weighted National Estimates, 2009-2019.

	2009[a]		2012		2016		2019	
	**N**	**%**	**N**	**%**	**N**	**%**	**N**	**%**
**Rural hospitals**	**228,714**		**156,167**		**80,705**		**62,729**	
Infant (up to 1 yr)	55,673	24.3 (23.7-25.0)	39,280	25.2 (24.3-26.0)	22,995	28.5 (27.0-30.0)	19,288	30.7 (28.6-32.9)
Early Childhood (1-4 yrs)	62,842	27.5 (26.7-28.2)	39,115	25.0 (24.2-25.9)	17,070	21.2 (20.0-22.3)	12,193	19.4 (18.2-20.7)
Middle Childhood (5-10 yrs)	35,828	15.7 (15.3-16.0)	24,374	15.6 (15.1-16.1)	10,949	13.6 (13.0-14.2)	7,537	12.0 (11.3-12.7)
Early Adolescence (11-14 yrs)	23,889	10.4 (10.0-10.9)	17,747	11.4 (10.7-12.0)	9,146	11.3 (10.3-12.4)	7,741	12.3 (10.9-13.8)
Later Adolescence (15-17 yrs)	50,483	22.1 (21.2-23.0)	35,651	22.8 (21.8-23.8)	20,545	25.5 (24.0-26.9)	15,969	25.5 (23.7-27.3)
**Urban non-teaching hospitals**	**572,723**		**378,216**		**177,222**		**92,118**	
Infant (up to 1 yr)	153,757	26.8 (24.3-29.4)	96,529	25.5 (24.5-26.5)	47,350	26.7 (25.1-28.4)	27,572	29.9 (27.5-32.4)
Early Childhood (1-4 yrs)	123,457	21.6 (20.4-22.7)	75,460	20.0 (19.1-20.8)	29,791	16.8 (15.4-18.2)	14,933	16.2 (14.5-17.9)
Middle Childhood (5-10 yrs)	82,516	14.4 (13.7-15.1)	55,006	14.5 (14.0-15.1)	24,395	13.8 (12.8-14.8)	10,827	11.8 (10.7-12.8)
Early Adolescence (11-14 yrs)	64,807	11.3 (10.7-12.0)	49,949	13.2 (12.4-14.0)	25,478	14.4 (13.0-15.8)	13,464	14.6 (12.7-16.6)
Later Adolescence (15-17 yrs)	148,187	25.9 (24.3-27.5)	101,272	26.8 (25.4-28.1)	50,208	28.3 (26.2-30.5)	25,322	27.5 (25.1-29.9)
**Urban teaching hospitals**	**960,824**		**1,013,348**		**1,001,976**		**1,002,652**	
Infant (up to 1 yr)	230,827	24.0 (23.3-24.7)	244,279	24.1 (23.5-24.7)	256,193	25.6 (24.6-26.5)	256,777	25.6 (24.8-26.4)
Early Childhood (1-4 yrs)	213,427	22.2 (21.6-22.8)	227,148	22.4 (21.8-23.0)	205,596	20.5 (19.9-21.2)	207,159	20.7 (19.9-21.4)
Middle Childhood (5-10 yrs)	167,084	17.4 (17.0-17.8)	178,958	17.7 (17.3-18.1)	172,318	17.2 (16.7-17.7)	161,760	16.1 (15.7-16.6)
Early Adolescence (11-14 yrs)	133,960	13.9 (13.5-14.3)	150,992	14.9 (14.5-15.3)	152,511	15.2 (14.7-15.7)	164,959	16.5 (15.9-17.0)
Later Adolescence (15-17 yrs)	215,526	22.4 (21.4-23.5)	211,971	20.9 (20.0-21.8)	215,358	21.5 (20.7-22.3)	211,997	21.1 (20.3-22.0)
**Freestanding children's hospitals**	**547,871**		**564,376**		**572,661**		**572,483**	
Infant (up to 1 yr)	153,545	28.0 (26.6-29.4)	152,509	27.0 (25.5-28.6)	154,449	27.0 (25.1-28.8)	153,059	26.7 (25.1-28.4)
Early Childhood (1-4 yrs)	139,604	25.5 (24.8-26.2)	139,923	24.8 (24.1-25.5)	134,491	23.5 (22.7-24.3)	137,729	24.1 (23.3-24.8)
Middle Childhood (5-10 yrs)	110,327	20.1 (19.6-20.7)	115,845	20.5 (19.9-21.1)	116,966	20.4 (19.7-21.2)	108,995	19.0 (18.4-19.7)
Early Adolescence (11-14 yrs)	81,311	14.8 (14.2-15.5)	87,365	15.5 (14.7-16.2)	90,729	15.8 (15.1-16.6)	93,789	16.4 (15.7-17.1)
Later Adolescence (15-17 yrs)	63,085	11.5 (10.7-12.3)	68,734	12.2 (11.4-12.9)	76,026	13.3 (12.5-14.1)	78,911	13.8 (13.1-14.5)

[a] Hospital type missing for 238,818 (3.7%) of hospitalizations in 2009

© 2023 Leyenaar JK et al. *JAMA Network Open.*

eTable 5. Complicated Birth Hospitalizations and Nonbirth Hospitalizations With Complex Chronic Diseases, Mental Health, and Disability Diagnoses, Weighted National Estimates, 2009-2019

	**2009**[a] Birth hospitalizations=3,856,059 Non-births=2,330,981	**2012** Birth hospitalizations=3,736,338 Non-births=2,121,092	**2016** Birth hospitalizations=3,770,047 Non-births=1,832,565	**2019** Birth hospitalizations=3,567,900 Non-births=1,729,982	**Relative % Change, 2009-2019**
**Complicated births,[b] n, % (95%CI)**	1,133,458 29.4% (28.8-29.9%)	1,115,917 29.9% (29.3-30.4%)	1,404,581 37.3% (36.6-37.9%)	1,497,243 42.0% (41.3-42.6%)	+42.8% (39.1; 46.5%)
Rural hospitals	119,132 25.1% (24.3-26.0%)	114,183 25.5% (24.7-26.3%)	116,267 31.2% (30.1-32.3%)	116,893 35.1% (34.0-36.2%)	+39.7% (32.7; 46.8%)
Urban non-teaching hospitals	473,013 27.4% (26.8-28.1%)	383,620 26.8% (26.2-27.5%)	306,781 33.0% (32.1-33.9%)	231,697 37.5% (36.4-38.6%)	+36.6% (30.6; 42.7%)
Urban teaching hospitals	536,676 32.6% (31.6-33.6%)	612,060 33.2% (32.3-34.1%)	969,733 39.6% (38.8-40.5%)	1,131,662 43.7% (42.9-44.6%)	+34.3% (29.6; 39%)
Freestanding children's hospitals	4,636 41.1% (18.9-63.4%)	6,055 46.4% (23.0-69.8%)	11,801 58.6% (44.3-72.9%)	16,992 56.1% (51.9-60.2%)	+36.3% (-19.4; 92.1%)
**Complex Chronic Disease Diagnosis,[c] n, % (95%CI)**	483,516 20.7% (19.6-21.9%)	519,672 24.5% (23.3-25.7%)	519,567 28.4% (27.0-29.7%)	522,168 30.2% (28.8-31.5%)	+45.5% (34.6; 56.4%)
Rural hospitals	16,306 7.1% (6.0-8.2%)	12,832 8.2% (6.8-9.7%)	6,684 8.3% (7.2-9.4%)	6,258 10.0% (8.1-11.9%)	+40.3% (-0.5; 81%)
Urban non-teaching hospitals	54,507 9.4% (8.3-10.5%)	39,062 10.2% (9.2-11.3%)	19,847 11.2% (9.8-12.6%)	9,910 10.8% (9.3-12.2%)	+14.7% (-6.6; 36.1%)
Urban teaching hospitals	224,413 23.1% (21.5-24.8%)	258,887, 25.4%(24.0-26.9%)	265,044 26.5% (25.1-27.8%)	272,922 27.2% (25.8-28.7%)	+17.6% (7.4; 27.8%)
Freestanding children's hospitals	188,290 34.2% (32.1-36.3%)	208,892 37.0% (34.8-39.2%)	227,992 39.8% (37.5-42.1%)	233,078 40.7% (38.4-43.0%)	+19.1% (9.1; 29.1%)
**Mental Health Diagnosis,[c] n, % (95%CI)**	325,975 14.0% (13.2-14.8%)	376,335 17.7% (16.8-18.7%)	399,902 21.8% (20.7-22.9%)	430,657 24.9% (23.7-26.1%)	+78.0% (61.6; 94.4%)
Rural hospitals	21,270 9.3% (8.1-10.4%)	18,406 11.8% (10.0-13.5%)	13,181 16.3% (13.4-19.3%)	12,632 20.1% (16.3-23.9%)	+117.1% (27.5; 206.6%)
Urban non-teaching hospitals	75,325 13.0% (11.2-14.7%)	68,481 17.9% (15.4-20.5%)	41,739 23.6% (19.2-27.9%)	23,441 25.4% (20.0-30.9%)	+96.4% (13.2; 179.6%)
Urban teaching hospitals	164,823 17.0% (15.7-18.3%)	201,711 19.8% (18.5-21.1%)	232,216 23.2% (21.7-24.7%)	268,763 26.8% (25.1-28.5%)	+57.7% (40.3; 75.1%)
Freestanding children's hospitals	64,558 11.7% (10.1-13.4%)	87,738 15.5% (13.7-17.4%)	112,767 19.7% (17.7-21.7%)	125,820 22.0% (20.1-23.9%)	+87.5% (53.9; 121.1%)
**Disability Diagnosis,[c] n, % (95%CI)**	249,089 10.7% (10.1-11.3%)	272,564 12.9% (12.2-13.5%)	308,908 16.9% (16.1-17.6%)	329,284 19.0% (18.2-19.9%)	+78.1% (63.1; 93.2%)
Rural hospitals	8,010 3.5%(2.9-4.1%)	6,392 4.1% (3.3-4.8%)	4,271 5.3% (4.5-6.1%)	4,128 6.6% (5.2-8.0%)	+88.3% (12.7; 164%)
Urban non-teaching hospitals	28,386 4.9% (4.4-5.4%)	21,586 5.7% (5.1-6.2%)	12,892 7.3% (6.4-8.1%)	6,653 7.2% (6.4-8.0%)	+47.9% (21.1; 74.7%)
Urban teaching hospitals	113,885 11.7% (10.9-12.6%)	133,733 13.1% (12.3-13.9%)	155,646 15.5% (14.7-16.4%)	172,820 17.2% (16.3-18.2%)	+46.8% (33.1; 60.4%)
Freestanding children's hospitals	98,808 17.9% (16.6-19.3%)	110,852 19.6% (18.3-20.9%)	136,099 23.8% (22.4-25.1%)	145,684 25.4% (24.1-26.8%)	+41.8% (28.6; 55.1%)

[a] Hospital type missing for 238,818 (3.7%) of hospitalizations in 2009, [b] denominator reflects all birth hospitalizations, [c] denominator reflects all non-birth hospitalizations

© 2023 Leyenaar JK et al. *JAMA Network Open.*
