# Supplementary material for: Pediatric Hospitalizations at Rural and Urban Teaching and Nonteaching Hospitals in the US, 2009-2019
Source: JAMA Netw Open. 2023 Sep 1;6(9):e2331807. doi: 10.1001/jamanetworkopen.2023.31807 (PMC10474556; doi:10.1001/jamanetworkopen.2023.31807)
Supplement: Supplement 2. — Data Sharing Statement [file jamanetwopen-e2331807-s002.pdf]

Data Sharing Statement

Leyenaar. Pediatric Hospitalizations at Rural and Urban Teaching and Nonteaching Hospitals in the US, 2009-2019. *JAMA Netw Open*. Published September 01, 2023. doi:10.1001/jamanetworkopen.2023.31807

Data
Data available: No

Additional Information
Explanation for why data not available: Our data use agreement prohibits data sharing.
